# Low Prevalance of Major Events Adverse to Exercise Stress Echocardiography

**DOI:** 10.5935/abc.20160096

**Published:** 2016-08

**Authors:** Stephanie Macedo Andrade, Caio José Coutinho Leal Telino, Antônio Carlos Sobral Sousa, Enaldo Vieira de Melo, Carla Carolina Cardoso Teixeira, Clarissa Karine Cardoso Teixeira, Jaquiele Santos Santana, Igor Larchert Mota, Carlos José Oliveira de Matos, Joselina Luzia Menezes Oliveira

**Affiliations:** 1 Universidade Federal de Sergipe, Aracaju, SE- Brazil; 2 Centro de Ensino e Pesquisa e Laboratório de Ecocardiografia (ECOLAB) do Hospital e Fundação São Lucas, Aracaju, SE - Brazil

**Keywords:** Coronary Artery Disease, Exercise / physiology, Exercise Test, Safety

## Abstract

**Background:**

Stress echocardiography is well validated for diagnosis and risk stratification of coronary artery disease. Exercise stress echocardiography (ESE) has been shown to be the most physiological among the modalities of stress, but its safety is not well established.

**Objective:**

To study the complications related to ESE and clinical and echocardiographic variables most commonly associated with their occurrence.

**Methods:**

Cross-sectional study consisting of 10250 patients submitted to ESE for convenience, from January 2000 to June 2014. Cardiac Arrhythmias (CA) were the most frequent complications observed during the examination. The volunteers were divided into two groups according to the occurrence of CA during ESE: G1 group, composed of patients who have CA, and G2 formed by individuals who did not show such complication. Results: Group G1, consisting of 2843 patients (27.7%), and Group G2 consisting of 7407 patients (72.3%). There was no death, acute myocardial infarction, ventricular fibrillation or asystole. Predominant CAs were: supraventricular extrasystoles (13.7%), and ventricular extrasystoles (11.5%). G1 group had a higher mean age, higher frequency of hypertension and smoking, larger aortic roots and left atrium (LA) and lower ejection fraction than G2. G1 group also had more ischemic changes (p < 0.001). The predictor variables were age (RR 1.04; [CI] 95% from 1.038 to 1.049) and LA (RR 1.64; [CI] 95% from 1.448 to 1.872).

**Conclusion:**

ESE proved to be a safe modality of stress, with non-fatal complications only. Advanced age and enlargement of the left atrium are predictive of cardiac arrhythmias.

## Introduction

Coronary artery disease (CAD) is the leading cause of morbidity and mortality in the world.^1,2^ In Brazil, cardiovascular diseases (CVD) are responsible for one third of deaths annually.^3^ Due to a large social impact, early examination of CAD has become mandatory to identify not only a high-risk group, in which additional interventions are necessary, but also to select a low-risk subgroup, in which additional procedures would not be required.^4,5^

Ergometric test (ET) is the non-invasive examination initially recommended for the diagnosis and stratification of risk in patients with suspected CAD.^6-8^ However, this method has limitations in certain situations, such as left bundle branch block or ST segment changes in resting electrocardiogram (ECG).^8^ Futhermore, changes in wall motion of the left ventricle detected through stress echocardiography (SE) appear earlier in the ischemic cascade than angina or ST segment changes, which gives SE better sensitivity and specificity.^9-12^

SE, which was introduced in the late 70's, initially as pharmacological stress testing, is the most well validated method for the diagnosis and stratification od risk, prognosis, and evaluation of myocardial viability in patients with CAD.^13-18^ Even though the use of drugs such stressors has proven safe, recent literature has firmly focused on its complications.^19-29^

Although both methods of testing yield similarly accurate results in patients with well-preserved physical capacity, Exercise stress echocardiography (ESE) is the better choice over pharmacological stress testing, with the latter being reserved to physically impaired individuals or those with low motivation to exercise.^30,31^ ESE is also a physiological and versatile method, whose use has been growing. However, literature on the method's safety and its complications is scarce.^32,33^ Therefore, the objective of this study is to analyse the complications related to ESE and the clinical and echocardiographical variables that are predictors of these events.

## Methods

This is an analytical and descriptive cross-sectional study conducted between January 2000 and June 2014

### Patients

The convenience sample was made up of 10250 patients with suspected or established CAD who were submitted to stress echocardiography through physical effort at the Echocardiography Laboratory of the *Clínica e Hospital São Lucas (ECOLAB)* in Aracaju, state of São Paulo. All patients over 25 years old, referred to the service by assisting doctors, were included, with the exception of those who refused to participate in the study. The recommendations for the exam were: pre-operative, typical or atypical chest pain, risk stratification, positive or negative ET, inefficient ET, check-up.

Patients were divided into two groups according to the occurrence of the most frequent complications observed in ESE: G1 group - patients who presented such complications in the exam, and G2 group - individuals with no complications.

### Clinical characteristics

Clinical data were collected and recorded through interviews conducted prior to the procedure. A structure questionnaire investigating weight, height, symptons such as dyspnea and chest pain, medications, CAD risk factors and history of personal or family heart disease was used, together with relevant data such as previous CADs such as acute myocardial infarction, percutaneous and surgical revascularization. In addition, previous results from lab work and examinations of the cardiovascular system were registered.

Obesity was characterized when body mass surpassed 30 kg/m^2^. Hypercholesterolemia was defined as total serum cholesterol levels of over 200 mg/dL (after fasting for 12 hours), and hypertriglyceridemia as serum triglycerides levels greater than 150 mg/dL (after fasting for 12 hours) or use of antilipêmico agent (statins and / or fibrates).

Systemic arterial hypertension was considered when blood pressure levels measured in the arm, in resting condition, were systolic blood pressure ≥ 140 mmHg and/or diastolic ≥ 90 mmHg, or when there was use of antihypertensive medication.

Diabetes mellitus was defined by the presence of fasting glucose over 126 mg/dL or by the use of insulin or oral hypoglycemic agents.

Old myocardial infarction was defined through clinical history and/or previous supplementary exams, such as ECG, echocardiogram and/or coronary angiography.

### Ergometric Test

Firstly, the protocol consisted of the 12-lead ECG and resting echocardiogram after clinical investigation. After that, physical effort on treadmill was performed, and soon after echocardiographic images were acquired again.

All patients were submitted to the standard Bruce or Ellestad protocols protocols during ergometric tests. There was continuous monitoring of heart rate, and the patients were encouraged to reach their maximum physical effort. For metabolic calculations, the volume of inspired oxygen at peak exercise (VO_2_max) was obtained indirectly through the following formula: VO_2_max = 14.76 - 1.379t + 0.451t^2^ - 0.012t^3^, in which t is the duration of the test^34^ in minutes. The load was expressed as metabolic equivalents, in which 1 MET corresponds to 3.5 mL/kg·min of inspired VO_2_, in resting.^35^ Throughout the test, the individuals were continuously monitored by 3-lead ECG.

Ischemic electrocardiographic changes were designated to the occurrence of descending or horizontal ST segment depression ≥ 1 mm for men and ≥ 1,5 mm for women at 0.08 seconds from the J point.^8^

### Exercise Stress Echocardiography

The environment for the performance of the exam is ergonomically projected by a constantly trained team, due to the hospital reputation as a reference hospital in the cardiology department and its level 3 accreditation by specific assessment. As a routine procedure, the suspension of beta-blockers three days before the exam is recommended, while other drugs are kept in use.

Exams were carried out with Hewlett Packard/Phillips SONOS 5500 up to 2012 and then with Phillips IE-33, with due observance of technical aspects described by Schiller et al.^36^ Bidimensional echocardiographic images were obtained at the parasternal and apical acoustic windows during resting and immediately after effort, with the patient in the left lateral decubitus and simultaneous electrocardiographic recording. The wall motion of the left ventricular (LV) wall was evaluated by experienced echocardiographer, with level III, as required by the American Echocardiography Society. Segmental wall thickening of the LV was quantitatively evaluated in resting and after physical effort through the 16 segment model with the following gradual rank: 1, normal; 2, hypokinetic; 3, akinetic; 4, dyskinetic. The left ventricular motion score index (LVMSI) was calculated in resting and during physical exercise as the sum of the scores given to each of the 16 segments divided by the number of segments evaluated at that moment. LVMSI of 1 corresponds to normality; from 1.1 to 1.6 corresponds to mild dysfunction; from 1.61 to 2, moderate dysfunction. Values of over 2 represent severe dysfunction.^36^ The difference between resting and physical exercise LVMSIs is called ΔLVMSI. The development of a new change in the parietal motility or worsening of existing dyssynergy (ΔLVMSI ≠ 0) were considered indicative of myocardial ischemia.

### Analysis of complication occurrence

Patients were monitored for possible complications by 12-lead ECG before, during and after physical exercise stress. The researched complications and their respective definitions were based on those described by Geleijnse et al.^29^

Major complications considered were: death, acute myocardial infarction, cardiac rupture, ventricular fibrillation and cardiac asystole.

Minor complications were defined as: atrioventricular block (AVB), coronary spasm, stroke, hypotension and hypertension at peak exercise, sustained ventricular tachycardia (> 30 beats / min), non-sustained ventricular tachycardia, sustained supraventricular tachycardia, non-sustained supraventricular tachycardia, ventricular premature beats and supraventricular extrasystoles.

### Statistical Analysis

Categorical variables were presented as percentages and analyzed using the chi-square test (c^2^) or Fisher's exact test. Continuous variables were represented by mean +/- standard deviation and compared with the aid of Student's unpaired t test or Mann-Whitney U test, as appropriate.

To assess the risk factors predictive of complications to ESE we used univariate and multivariate Cox regression analysis. The variables included in the multivariate model were all of those with p < 0.1 in the univariate analysis. Multicollinearity problems were resolved prior to insertion of the variables in the model. Forward selection of variables was used. Variables that remained in the model were tested for possible interactions. Proportional hazards assumption was tested by Schoenfeld residual testing for each of the variables that remained in the final model. Values of p < 0.05 were considered significant. Statistical analysis were processed by the software *Statistical Package for the Social Sciences* (*SPSS*) 21.0 (Chicago, IL).

### Ethical aspects

The ethical principles for human experimentation were followed thoroughly, and all patients signed an informed consent. The study was approved by the Ethics Committee of the Federal University of Sergipe (CAAE 1818.0.000.107-06).

## Results

A total of 10250 patients participated in the study; 4936 males (48.2%), with a mean age of 57.19 ± 11.24 years, limited to 25 and 93 years of age. There was no record of major complications such as death, acute myocardial infarction, cardiac rupture, ventricular fibrillation and cardiac asystole.

Cardiac arrhythmias were the most frequent observed complication during the exam, and thus the distribution of the groups was as follows: 2843 patients in the G1 group (27.7%), and 7407 patients in the G2 group (72.3%).

One case of each of the following complications was recorded: AVB; coronary spasm; stroke; and arterial hypotension, which represented 0.009% of the sample. Arterial hypertension at the peak of physical effort was observed in 1011 individuals (9.9% of the population), of which 82% presented with systemic arterial hypertension (SAH) before the exam.

The most frequently observed complications in this study were cardiac arrhythmias. The observed rates were non-sustained ventricular and supraventricular tachycardia and ventricular and supraventricular extrasystoles be it isolated, in saves, bigeminal, monomorphic or polymorphic. Their frequency is described in Table 1. In all cases they were reversed with the cessation of physical effort and use of isosorbide dinitrate and inhaled oxygen when in the presence of associated myocardial ischemia, with no hemodynamic repercussions that would require ICU admission, considering that ventricular fibrillation or sustained ventricular tachycardia were not recorded.

**Table 1:** Frequency of cardiac arrhythmias during ESE

Cardiac Arrhythmia	Frequency n = 10250
Sustained ventricular tachycardia	0 (0%)
Non-sustained ventricular tachycardia	114 (1.1%)
Sustained supraventricular tachycardia	0 (0%)
Non-sustained supraventricular tachycardia	645 (6.2%)
Ventricular extrasystoles	1186 (11.5%)
Supraventricular extrasystoles	1418 (13.7%)

ESE: exercise stress echocardiography

The 10250 patients were divided into two groups: G1 group - made up of 2843 patients (27.7%) that presented with cardiac arrhythmias during the exam, and G2 group - made up of 7404 patients (72.3%) with no arrhythmias.

### Clinical characteristics of the groups

G1 group presented higher mean age, higher SAH frequency and smoking. With regoards to gender and other associated comorbidities, both groups presented similar frequencies (Table 2)

**Tabela 2:** Clinical characteristics of patients with (G1) or without (G2) cardiac arrhythmias during ESE

Clinical characteristics	G1 n = 2843 (27.7%)	G2 n = 7407 (72.2%)	p
Male	1410 (49.6%)	3525 (47.6%)	0.06
Age (mean in years)	60.81 ± 10.78	55.79 ± 11.1	< 0.001
BMI (kg/m^2^)	27.2 ± 4.28	27.3 ± 4.43	0.3
SAH	1828 (64.3%)	4251 (57.4%)	< 0.001
Diabetes Mellitus	102 (3.6%)	637 (8.6%)	0.12
Dyslipidemia	1709 (60.1%)	4370 (59%)	0.33
Smoking	114 (4%)	378 (5.1%)	0.02
Family history of heart disease	1595 (56.1%)	4163 (56.2%)	0.92
Dyspnea	264 (9.3%)	555 (7.5%)	0.003
Precordialgia	816 (28.7%)	2326 (31.4%)	0.191
Previous AMI	165 (5.8%)	318 (4.3%)	0.002
Angioplasty	213 (7.5%)	533 (7.2%)	0.639
Myocardial revascularization	205 (7.2%)	385 (5.2%)	< 0.001
Beta-blocker	690 (24.3%)	1555 (21.0%)	< 0.001
Calcium channel blockers	239 (8.4%)	481 (6.5%)	0.001
IACE	318 (11.2%)	674 (9.1%)	0.001
ARB	424 (14.9%)	1059 (14.3%)	0.468
Statin	640 (22.5%)	1504 (20.3%)	0.016
Glycemia	100.03 ± 25.26	99.72 ± 29.23	0.907
Total cholesterol (mg/dL)	196.86 ± 47.81	192.88 ± 46.28	0.364
HDL (mg/dL)	49.95 ± 13.97	46.54 ± 13.33	0.748
LDL (mg/dL)	123.28 ± 42.16	116.87 ± 41.28	0.115
Triglycerides (mg/dL)	148.95 ± 89.06	154.78 ± 100.74	0.535

BMI: body mass index; SAH: systemic arterial hypertension; AMI: acute myocardial infarction; IACE: inhibitor of angiotensin converting enzyme; ARB: angiotensin receptor blocker; HDL: hight density lipoproteins, LDL: low density lipoproteins.

### Echocardiographic characteristics of the groups

In the transthoracic Doppler echocardiography there was significant difference in the diameters of the aorta and the left atrium and in the left ventricular mass and thickness. Ejection fraction of the LV was preserved in both groups, which was lower in G1 patients (Table 3).

**Table 3:** Echocardiographic characteristis of patients with (G1) or without (G2) cardiac arrhythmia during ESE

Echocardiographic characteristis		G1 n = 2843 (27,7%)	G2 n = 7407 (72,3%)	p
Aorta (cm)		3.23 ± 0.39	3.17 ± 0.4	< 0.001
LA (cm)		3.87 ± 0.47	3.76 ± 0.43	< 0.001
LA volume (ml/m^2^)		24.26 ± 24.25	22.52 ± 26.02	0.11
E/e' ratio		9.94 ± 3.4	9.41 ± 3.36	0.005
LV mass index (g/m^2^)		95.2 ± 87.74	97.9 ± 62.88	< 0.001
Relative thickness of LV wall		31.9 ± 5.42	32.3 ± 6.77	0.005
Ejection fraction (%)		0.66 ± 0.06	0.67 ± 0.06	< 0.001
Resting LVMSI		1.039 ± 0.141	1.022 ± 0.104	< 0.001
Physical effort LVMSI		1.056 ± 0.153	1.035 ± 0.117	< 0.001
**ESE Results **				
	– Normal	71.4%	78.8%	< 0.001
	– Ischemic	12.8%	10%	
	– Fixed ischemia	10.9%	8.7%	
	– Fixed and induced ischemia	4.9%	2.4%	

LA: left atrium; LV: left ventricle; LVMSI: left ventricle motion score index; ESE: exercise stress echocardiography.

In the ESE, there was a difference in the left ventricular motion score index in resting and during physical effort. There was also frequent presence of myocardial ischemia in G1 patients.

### Logistic regression analysis

The test to determine the presence of possible confounders in the model showed that only two variables were independently associated to the occurrence of complications. There was no interaction between the analysed variables (Table 4).

**Table 4:** Multivariate logistic regression with parameters associated to the presence of cardiac arrhythmias during ESE

Variable	Odds Ratio	CI95%	p
Age	1.04	1.038 – 1.049	< 0.001
Left Atrium	1.64	1.448 – 1.872	< 0.001

CI: confidence interval; LA: left atrium; ESE: exercise stress echocardiography.

## Discussion

ESE proved to be safe in this study, with low frequency of complications, none of which led to death. Death is more frequently observed in SE than in pharmacological stress testing.^29^ This may be due to the mechanism through which stressor drugs act in the heart: dobutamine stimulates adrenergic receptors, increasing chronotropism and inotropism, and with great arrhythmogenic and hypertensive potential; dipyridamole stresses the heart muscle by stimulating adenosine receptors and reducing subendocardial flow - it also has a negative chronotropic and dromotropic effect as well as bronchoconstrictor activity. However, physical exercise represents a more physiological modality of stress free of the side effects described above.^18^ There is also a selection bias: candidates eligible for the test physical effort present with preserved ejection fraction, while pharmacological stress testing patients may have more advanced heart diseases.

In a large multicenter study with 85,997 patients undergoing SE - 26,295 of which were tested by physical stress and the others by pharmacological stress - there was no death outcome in the group tested by ESE. In the pharmacological stress group, however, five deaths were observed. With regards to the frequency of life threatening events, the proportion of the in the ESE group was 1 in 6574, whereas in the group receiving dipyridamole and dobutamine, we saw 1 in every 1294, and 1 in every 557, respectively.^32^

In a Moroccan study with 311 patients, 206 of whom were submitted to ESE, no major or lethal events were registered.^33^ According to Sicari et al.^18^ pharmacological stress from dobutamine triggers severe complications in 3 of 1,000 patients, and pharmacological stress from dipyridamole triggers severe complications in 1 of 1,000 patients.

The most frequente minor complications were arrhythmias, especially those with less hemodynamic repercussions and easily reversible upon cessation of effort, such as supraventricular and ventricular extrasystoles. This is according to one study and is not mentioned in other bigger studies.^29,32^

Arterial hypertension due to effort was observed in a significant portion of the sample (9.9% of cases) mostly in chronic hypertensive individuals. One of the reasons for this marked decompensation in the pressure response to stress may be the protocol for exam preparation of our institution, which includes a suspention of beta-blockers for three days prior to the exam. There was a significant difference considering the available literature, which may be because most studies use pharmacological stress, which may have severe hypotension and cardiogenic shock as a response in up to 7.6% of cases.^29,32^

The clinical and demographic profile of the sample is only mentioned by Fennich et al.^33^ In this study, age was similar to that of this paper (59.2 years of age). However, diabetes mellitus and smoking were more prevalent and SAH was lower, differently from our findings.

The predictor independent variables, left atrium, and age presented a close relationship with each other. Some studies have reported that the size of the left atrium would naturally increase with age.^37-39^ Thus, senescence would provide changes that would culminate with left atrium dilatation and dysfunction, thus increasing predisposition to atrial arrhythmias,^39^ which may influence their occurrence when the patient is submitted to stress, as in ESE. Still with regards to age, this finding as an independent predictor of complications during ESE is very important, considering this is a commonly observed fact in clinical practice. However, it is not yet described in relation to physical effort in literature.

Another gap in the literature concerns the relation between the echocardiographic variables and the occurrence of complications during ESE, considering that our study found several significant differences, and there are no studies about them. Another relevant observation is that of a very significant difference in the presence of myocardial ischemia in patients who had adverse events during ESE, maybe due to coronary arterial disease, which alters the cardiac electrical conduction system and induces arrhythmias; a frequently studied event during pharmacological stress, included in the work of Abreu et al.^40^ but still without supporting evidence for ESE.

Although current guidelines^41^ recommend the use of this methodology in moderate-risk patients, in clinical practice we often see it used in high-risk individuals. However, even in those cases, no identifiable complications were found.

This study shows us interesting data that is still scarcely study in the field of ESE - a method that has been growing due to its versatility, reproducibility, and cost-effectiveness. We expect that this growth prompt new studies around this theme.

About the limitations of this study, we would highlight those applicable to any observational study, in which unmeasured variables may corroborate the statistical difference between the groups. In this sample, we also find a pre-selection bias, since patients submitted to ESE presented with based heart diseases or fisk factors to their development.

## Conclusion

ESE is a safe stress modality, with no risk-of-death complications observed. Adverse events recorded were minor and easily reversed by cessation of physical effort. The safety and feasibility of this stress mode associated with its important diagnostic and prognostic validity, justify its use in clinical practice.
